# The Importance of Students’ Motivation for Their Academic Achievement – Replicating and Extending Previous Findings

**DOI:** 10.3389/fpsyg.2019.01730

**Published:** 2019-07-31

**Authors:** Ricarda Steinmayr, Anne F. Weidinger, Malte Schwinger, Birgit Spinath

**Affiliations:** ^1^Department of Psychology, TU Dortmund University, Dortmund, Germany; ^2^Department of Psychology, Philipps-Universität Marburg, Marburg, Germany; ^3^Department of Psychology, Heidelberg University, Heidelberg, Germany

**Keywords:** academic achievement, ability self-concept, task values, goals, achievement motives, intelligence, relative weight analysis

## Abstract

Achievement motivation is not a single construct but rather subsumes a variety of different constructs like ability self-concepts, task values, goals, and achievement motives. The few existing studies that investigated diverse motivational constructs as predictors of school students’ academic achievement above and beyond students’ cognitive abilities and prior achievement showed that most motivational constructs predicted academic achievement beyond intelligence and that students’ ability self-concepts and task values are more powerful in predicting their achievement than goals and achievement motives. The aim of the present study was to investigate whether the reported previous findings can be replicated when ability self-concepts, task values, goals, and achievement motives are all assessed at the same level of specificity as the achievement criteria (e.g., hope for success in math and math grades). The sample comprised 345 11th and 12th grade students (*M* = 17.48 years old, *SD* = 1.06) from the highest academic track (Gymnasium) in Germany. Students self-reported their ability self-concepts, task values, goal orientations, and achievement motives in math, German, and school in general. Additionally, we assessed their intelligence and their current and prior Grade point average and grades in math and German. Relative weight analyses revealed that domain-specific ability self-concept, motives, task values and learning goals but not performance goals explained a significant amount of variance in grades above all other predictors of which ability self-concept was the strongest predictor. Results are discussed with respect to their implications for investigating motivational constructs with different theoretical foundation.

## Introduction

Achievement motivation energizes and directs behavior toward achievement and therefore is known to be an important determinant of academic success (e.g., Robbins et al., 2004; Hattie, 2009; Plante et al., 2013; Wigfield et al., 2016). Achievement motivation is not a single construct but rather subsumes a variety of different constructs like motivational beliefs, task values, goals, and achievement motives (see Murphy and Alexander, 2000; Wigfield and Cambria, 2010; Wigfield et al., 2016). Nevertheless, there is still a limited number of studies, that investigated (1) diverse motivational constructs in relation to students’ academic achievement in one sample and (2) additionally considered students’ cognitive abilities and their prior achievement (Steinmayr and Spinath, 2009; Kriegbaum et al., 2015). Because students’ cognitive abilities and their prior achievement are among the best single predictors of academic success (e.g., Kuncel et al., 2004; Hailikari et al., 2007), it is necessary to include them in the analyses when evaluating the importance of motivational factors for students’ achievement. Steinmayr and Spinath (2009) did so and revealed that students’ domain-specific ability self-concepts followed by domain-specific task values were the best predictors of students’ math and German grades compared to students’ goals and achievement motives. However, a flaw of their study is that they did not assess all motivational constructs at the same level of specificity as the achievement criteria. For example, achievement motives were measured on a domain-general level (e.g., “Difficult problems appeal to me”), whereas students’ achievement as well as motivational beliefs and task values were assessed domain-specifically (e.g., math grades, math self-concept, math task values). The importance of students’ achievement motives for math and German grades might have been underestimated because the specificity levels of predictor and criterion variables did not match (e.g., Ajzen and Fishbein, 1977; Baranik et al., 2010). The aim of the present study was to investigate whether the seminal findings by Steinmayr and Spinath (2009) will hold when motivational beliefs, task values, goals, and achievement motives are all assessed at the same level of specificity as the achievement criteria. This is an important question with respect to motivation theory and future research in this field. Moreover, based on the findings it might be possible to better judge which kind of motivation should especially be fostered in school to improve achievement. This is important information for interventions aiming at enhancing students’ motivation in school.

### Theoretical Relations Between Achievement Motivation and Academic Achievement

We take a social-cognitive approach to motivation (see also Pintrich et al., 1993; Elliot and Church, 1997; Wigfield and Cambria, 2010). This approach emphasizes the important role of students’ beliefs and their interpretations of actual events, as well as the role of the achievement context for motivational dynamics (see Weiner, 1992; Pintrich et al., 1993; Wigfield and Cambria, 2010). Social cognitive models of achievement motivation (e.g., expectancy-value theory by Eccles and Wigfield, 2002; hierarchical model of achievement motivation by Elliot and Church, 1997) comprise a variety of motivation constructs that can be organized in two broad categories (see Pintrich et al., 1993, p. 176): students’ “beliefs about their capability to perform a task,” also called expectancy components (e.g., ability self-concepts, self-efficacy), and their “motivational beliefs about their reasons for choosing to do a task,” also called value components (e.g., task values, goals). The literature on motivation constructs from these categories is extensive (see Wigfield and Cambria, 2010). In this article, we focus on selected constructs, namely students’ ability self-concepts (from the category “expectancy components of motivation”), and their task values and goal orientations (from the category “value components of motivation”).

According to the social cognitive perspective, students’ motivation is relatively situation or context specific (see Pintrich et al., 1993). To gain a comprehensive picture of the relation between students’ motivation and their academic achievement, we additionally take into account a traditional personality model of motivation, the theory of the achievement motive (McClelland et al., 1953), according to which students’ motivation is conceptualized as a relatively stable trait. Thus, we consider the achievement motives hope for success and fear of failure besides students’ ability self-concepts, their task values, and goal orientations in this article. In the following, we describe the motivation constructs in more detail.

Students’ ability self-concepts are defined as cognitive representations of their ability level (Marsh, 1990; Wigfield et al., 2016). Ability self-concepts have been shown to be domain-specific from the early school years on (e.g., Wigfield et al., 1997). Consequently, they are frequently assessed with regard to a certain domain (e.g., with regard to school in general vs. with regard to math).

In the present article, task values are defined in the sense of the expectancy-value model by Eccles et al. (1983) and Eccles and Wigfield (2002). According to the expectancy-value model there are three task values that should be positively associated with achievement, namely intrinsic values, utility value, and personal importance (Eccles and Wigfield, 1995). Because task values are domain-specific from the early school years on (e.g., Eccles et al., 1993; Eccles and Wigfield, 1995), they are also assessed with reference to specific subjects (e.g., “How much do you like math?”) or on a more general level with regard to school in general (e.g., “How much do you like going to school?”).

Students’ goal orientations are broader cognitive orientations that students have toward their learning and they reflect the reasons for doing a task (see Dweck and Leggett, 1988). Therefore, they fall in the broad category of “value components of motivation.” Initially, researchers distinguished between learning and performance goals when describing goal orientations (Nicholls, 1984; Dweck and Leggett, 1988). Learning goals (“task involvement” or “mastery goals”) describe people’s willingness to improve their skills, learn new things, and develop their competence, whereas performance goals (“ego involvement”) focus on demonstrating one’s higher competence and hiding one’s incompetence relative to others (e.g., Elliot and McGregor, 2001). Performance goals were later further subdivided into performance-approach (striving to demonstrate competence) and performance-avoidance goals (striving to avoid looking incompetent, e.g., Elliot and Church, 1997; Middleton and Midgley, 1997). Some researchers have included work avoidance as another component of achievement goals (e.g., Nicholls, 1984; Harackiewicz et al., 1997). Work avoidance refers to the goal of investing as little effort as possible (Kumar and Jagacinski, 2011). Goal orientations can be assessed in reference to specific subjects (e.g., math) or on a more general level (e.g., in reference to school in general).

McClelland et al. (1953) distinguish the achievement motives hope for success (i.e., positive emotions and the belief that one can succeed) and fear of failure (i.e., negative emotions and the fear that the achievement situation is out of one’s depth). According to McClelland’s definition, need for achievement is measured by describing affective experiences or associations such as fear or joy in achievement situations. Achievement motives are conceptualized as being relatively stable over time. Consequently, need for achievement is theorized to be domain-general and, thus, usually assessed without referring to a certain domain or situation (e.g., Steinmayr and Spinath, 2009). However, Sparfeldt and Rost (2011) demonstrated that operationalizing achievement motives subject-specifically is psychometrically useful and results in better criterion validities compared with a domain-general operationalization.

### Empirical Evidence on the Relative Importance of Achievement Motivation Constructs for Academic Achievement

A myriad of single studies (e.g., Linnenbrink-Garcia et al., 2018; Muenks et al., 2018; Steinmayr et al., 2018) and several meta-analyses (e.g., Robbins et al., 2004; Möller et al., 2009; Hulleman et al., 2010; Huang, 2011) support the hypothesis of social cognitive motivation models that students’ motivational beliefs are significantly related to their academic achievement. However, to judge the relative importance of motivation constructs for academic achievement, studies need (1) to investigate diverse motivational constructs in one sample and (2) to consider students’ cognitive abilities and their prior achievement, too, because the latter are among the best single predictors of academic success (e.g., Kuncel et al., 2004; Hailikari et al., 2007). For effective educational policy and school reform, it is crucial to obtain robust empirical evidence for whether various motivational constructs can explain variance in school performance over and above intelligence and prior achievement. Without including the latter constructs, we might overestimate the importance of motivation for achievement. Providing evidence that students’ achievement motivation is incrementally valid in predicting their academic achievement beyond their intelligence or prior achievement would emphasize the necessity of designing appropriate interventions for improving students’ school-related motivation.

There are several studies that included expectancy and value components of motivation as predictors of students’ academic achievement (grades or test scores) and additionally considered students’ prior achievement (Marsh et al., 2005; Steinmayr et al., 2018, Study 1) or their intelligence (Spinath et al., 2006; Lotz et al., 2018; Schneider et al., 2018; Steinmayr et al., 2018, Study 2, Weber et al., 2013). However, only few studies considered intelligence and prior achievement together with more than two motivational constructs as predictors of school students’ achievement (Steinmayr and Spinath, 2009; Kriegbaum et al., 2015). Kriegbaum et al. (2015) examined two expectancy components (i.e., ability self-concept and self-efficacy) and eight value components (i.e., interest, enjoyment, usefulness, learning goals, performance-approach, performance-avoidance goals, and work avoidance) in the domain of math. Steinmayr and Spinath (2009) investigated the role of an expectancy component (i.e., ability self-concept), five value components (i.e., task values, learning goals, performance-approach, performance-avoidance goals, and work avoidance), and students’ achievement motives (i.e., hope for success, fear of failure, and need for achievement) for students’ grades in math and German and their GPA. Both studies used relative weights analyses to compare the predictive power of all variables simultaneously while taking into account multicollinearity of the predictors (Johnson and LeBreton, 2004; Tonidandel and LeBreton, 2011). Findings showed that – after controlling for differences in students‘ intelligence and their prior achievement – expectancy components (ability self-concept, self-efficacy) were the best motivational predictors of achievement followed by task values (i.e., intrinsic/enjoyment, attainment, and utility), need for achievement and learning goals (Steinmayr and Spinath, 2009; Kriegbaum et al., 2015). However, Steinmayr and Spinath (2009) who investigated the relations in three different domains did not assess all motivational constructs on the same level of specificity as the achievement criteria. More precisely, students’ achievement as well as motivational beliefs and task values were assessed domain-specifically (e.g., math grades, math self-concept, math task values), whereas students’ goals were only measured for school in general (e.g., “In school it is important for me to learn as much as possible”) and students’ achievement motives were only measured on a domain-general level (e.g., “Difficult problems appeal to me”). Thus, the importance of goals and achievement motives for math and German grades might have been underestimated because the specificity levels of predictor and criterion variables did not match (e.g., Ajzen and Fishbein, 1977; Baranik et al., 2010). Assessing students’ goals and their achievement motives with reference to a specific subject might result in higher associations with domain-specific achievement criteria (see Sparfeldt and Rost, 2011).

Taken together, although previous work underlines the important roles of expectancy and value components of motivation for school students’ academic achievement, hitherto, we know little about the relative importance of expectancy components, task values, goals, and achievement motives in different domains when all of them are assessed at the same level of specificity as the achievement criteria (e.g., achievement motives in math → math grades; ability self-concept for school → GPA).

### The Present Research

The goal of the present study was to examine the relative importance of several of the most important achievement motivation constructs in predicting school students’ achievement. We substantially extend previous work in this field by considering (1) diverse motivational constructs, (2) students’ intelligence and their prior achievement as achievement predictors in one sample, and (3) by assessing all predictors on the same level of specificity as the achievement criteria. Moreover, we investigated the relations in three different domains: school in general, math, and German. Because there is no study that assessed students’ goal orientations and achievement motives besides their ability self-concept and task values on the same level of specificity as the achievement criteria, we could not derive any specific hypotheses on the relative importance of these constructs, but instead investigated the following research question (RQ):

RQ. What is the relative importance of students’ domain-specific ability self-concepts, task values, goal orientations, and achievement motives for their grades in the respective domain when including all of them, students’ intelligence and prior achievement simultaneously in the analytic models?

## Materials and Methods

### Participants and Procedure

A sample of 345 students was recruited from two German schools attending the highest academic track (Gymnasium). Only 11th graders participated at one school, whereas 11th and 12th graders participated at the other. Students of the different grades and schools did not differ significantly on any of the assessed measures. Students represented the typical population of this type of school in Germany; that is, the majority was Caucasian and came from medium to high socioeconomic status homes. At the time of testing, students were on average 17.48 years old (*SD* = 1.06). As is typical for this kind of school, the sample comprised more girls (*n* = 200) than boys (*n* = 145). We verify that the study is in accordance with established ethical guidelines. Approval by an ethics committee was not required as per the institution’s guidelines and applicable regulations in the federal state where the study was conducted. Participation was voluntarily and no deception took place. Before testing, we received written informed consent forms from the students and from the parents of the students who were under the age of 18 on the day of the testing. If students did not want to participate, they could spend the testing time in their teacher’s room with an extra assignment. All students agreed to participate. Testing took place during regular classes in schools in 2013. Tests were administered by trained research assistants and lasted about 2.5 h. Students filled in the achievement motivation questionnaires first, and the intelligence test was administered afterward. Before the intelligence test, there was a short break.

### Measures

#### Ability Self-Concept

Students’ ability self-concepts were assessed with four items per domain (Schöne et al., 2002). Students indicated on a 5-point scale ranging from 1 (totally disagree) to 5 (totally agree) how good they thought they were at different activities in school in general, math, and German (“I am good at school in general/math/German,” “It is easy to for me to learn in school in general/math/German,” “In school in general/math/German, I know a lot,” and “Most assignments in school/math/German are easy for me”). Internal consistency (Cronbach’s α) of the ability self-concept scale was high in school in general, in math, and in German (0.82 ≤ α ≤ 0.95; see Table 1).

**TABLE 1:** Means (*M*), Standard Deviations (*SD*), and Reliabilities (α) for all measures.

**Domain**	**School**			**Math**			**German**			**Intelligence**		
Variables	*M*	*SD*	*α*	*M*	*SD*	*α*	*M*	*SD*	*α*	*M*	*SD*	*α*
ASC	3.53	0.54	0.82	3.26	1.01	0.95	3.59	0.82	0.92			
Task values	3.72	0.68	0.90	3.38	0.90	0.93	3.67	0.79	0.92			
LG	3.83	0.58	0.83	3.65	0.77	0.88	3.77	0.67	0.86			
P-ApG	2.49	0.82	0.85	3.12	0.84	0.88	2.46	0.81	0.85			
P-AvG	3.24	0.75	0.89	2.41	0.81	0.89	3.17	0.77	0.89			
WA	2.60	0.85	0.91	2.61	0.90	0.91	2.64	0.87	0.92			
HfS	2.71	0.61	0.88	2.65	0.79	0.92	2.64	0.68	0.91			
FoF	1.95	0.66	0.90	1.99	0.71	0.90	1.88	0.68	0.91			
Grade	4.13	0.67		3.98	1.11		4.16	0.87				
g										108.84	17.76	0.90
Numerical										34.59	6.09	0.89
Verbal										40.15	9.38	0.71

**N* = 345 students. ASC, ability self-concept; LG, learning goals; P-ApG, performance-approach goals; P-AvG, performance-avoidance goals; WA, work avoidance; HfS, hope for success; FoF, fear of failure; g, general intelligence; Numerical, numeric intelligence; Verbal, verbal intelligence. Grades were recoded.*

#### Task Values

Students’ task values were assessed with an established German scale (SESSW; Subjective scholastic value scale; Steinmayr and Spinath, 2010). The measure is an adaptation of items used by Eccles and Wigfield (1995) in different studies. It assesses intrinsic values, utility, and personal importance with three items each. Students indicated on a 5-point scale ranging from 1 (totally disagree) to 5 (totally agree) how much they valued school in general, math, and German (Intrinsic values: “I like school/math/German,” “I enjoy doing things in school/math/German,” and “I find school in general/math/German interesting”; Utility: “How useful is what you learn in school/math/German in general?,” “School/math/German will be useful in my future,” “The things I learn in school/math/German will be of use in my future life”; Personal importance: “Being good at school/math/German is important to me,” “To be good at school/math/German means a lot to me,” “Attainment in school/math/German is important to me”). Internal consistency of the values scale was high in all domains (0.90 ≤ α ≤ 0.93; see Table 1).

#### Goal Orientations

Students’ goal orientations were assessed with an established German self-report measure (SELLMO; Scales for measuring learning and achievement motivation; Spinath et al., 2002). In accordance with Sparfeldt et al. (2007), we assessed goal orientations with regard to different domains: school in general, math, and German. In each domain, we used the SELLMO to assess students’ learning goals, performance-avoidance goals, and work avoidance with eight items each and their performance-approach goals with seven items. Students’ answered the items on a 5-point scale ranging from 1 (totally disagree) to 5 (totally agree). All items except for the work avoidance items are printed in Spinath and Steinmayr (2012), p. 1148). A sample item to assess work avoidance is: “In school/math/German, it is important to me to do as little work as possible.” Internal consistency of the learning goals scale was high in all domains (0.83 ≤ α ≤ 0.88). The same was true for performance-approach goals (0.85 ≤ α ≤ 0.88), performance-avoidance goals (α = 0.89), and work avoidance (0.91 ≤ α ≤ 0.92; see Table 1).

#### Achievement Motives

Achievement motives were assessed with the Achievement Motives Scale (AMS; Gjesme and Nygard, 1970; Göttert and Kuhl, 1980). In the present study, we used a short form measuring “hope for success” and “fear of failure” with the seven items per subscale that showed the highest factor loadings. Both subscales were assessed in three domains: school in general, math, and German. Students’ answered all items on a 4-point scale ranging from 1 (does not apply at all) to 4 (fully applies). An example hope for success item is “In school/math/German, difficult problems appeal to me,” and an example fear of failure item is “In school/math/German, matters that are slightly difficult disconcert me.” Internal consistencies of hope for success and fear of failure scales were high in all domains (hope for success: 0.88 ≤ α ≤ 0.92; fear of failure: 0.90 ≤ α ≤ 0.91; see Table 1).

#### Intelligence

Intelligence was measured with the basic module of the Intelligence Structure Test 2000 R, a well-established German multifactor intelligence measure (I-S-T 2000 R; Amthauer et al., 2001). The basic module of the test offers assessments of domain-specific intelligence for verbal, numeric, and figural abilities as well as an overall intelligence score (a composite of the three facets). The overall intelligence score is thought to measure reasoning as a higher order factor of intelligence and can be interpreted as a measure of general intelligence, *g*. Its construct validity has been demonstrated in several studies (Amthauer et al., 2001; Steinmayr and Amelang, 2006). In the present study, we used the scores that were closest to the domains we investigated: overall intelligence, numerical intelligence, and verbal intelligence (see also Steinmayr and Spinath, 2009). Raw values could range from 0 to 60 for verbal and numerical intelligence, and from 0 to 180 for overall intelligence. Internal consistencies of all intelligence scales were high (0.71 ≤ α ≤ 0.90; see Table 1).

#### Academic Achievement

For all students, the school delivered the report cards that the students received 3 months before testing (t0) and 4 months after testing (t2), at the end of the term in which testing took place. We assessed students’ grades in German and math as well as their overall grade point average (GPA) as criteria for school performance. GPA was computed as the mean of all available grades, not including grades in the nonacademic domains Sports and Music/Art as they did not correlate with the other grades. Grades ranged from 1 to 6, and were recoded so that higher numbers represented better performance.

### Statistical Analyses

We conducted relative weight analyses to predict students’ academic achievement separately in math, German, and school in general. The relative weight analysis is a statistical procedure that enables to determine the relative importance of each predictor in a multiple regression analysis (“relative weight”) and to take adequately into account the multicollinearity of the different motivational constructs (for details, see Johnson and LeBreton, 2004; Tonidandel and LeBreton, 2011). Basically, it uses a variable transformation approach to create a new set of predictors that are orthogonal to one another (i.e., uncorrelated). Then, the criterion is regressed on these new orthogonal predictors, and the resulting standardized regression coefficients can be used because they no longer suffer from the deleterious effects of multicollinearity. These standardized regression weights are then transformed back into the metric of the original predictors. The rescaled relative weight of a predictor can easily be transformed into the percentage of variance that is uniquely explained by this predictor when dividing the relative weight of the specific predictor by the total variance explained by all predictors in the regression model (*R*^2^). We performed the relative weight analyses in three steps. In Model 1, we included the different achievement motivation variables assessed in the respective domain in the analyses. In Model 2, we entered intelligence into the analyses in addition to the achievement motivation variables. In Model 3, we included prior school performance indicated by grades measured before testing in addition to all of the motivation variables and intelligence. For all three steps, we tested for whether all relative weight factors differed significantly from each other (see Johnson, 2004) to determine which motivational construct was most important in predicting academic achievement (RQ).

## Results

### Descriptive Statistics and Intercorrelations

Table 1 shows means, standard deviations, and reliabilities. Tables 2–4 show the correlations between all scales in school in general, in math, and in German. Of particular relevance here, are the correlations between the motivational constructs and students’ school grades. In all three domains (i.e., school in general/math/German), out of all motivational predictor variables, students’ ability self-concepts showed the strongest associations with subsequent grades (*r* = 0.53/0.61/0.46; see Tables 2–4). Except for students’ performance-avoidance goals (−0.04 ≤ *r* ≤ 0.07, *p* > 0.05), the other motivational constructs were also significantly related to school grades. Most of the respective correlations were evenly dispersed around a moderate effect size of |*r* | = 0.30.

**TABLE 2:** Intercorrelations between all variables in school in general.

**Motivation in school**	**Task values**	**LG**	**P-AgG**	**P-AvG**	**WA**	**HfS**	**FoF**	g	**GPAt0**	**GPAt2**
ASC	0.45	0.41	0.00	0.29	−0.27	0.45	−0.31	0.13	0.53	**0.53**
Task Values		0.57	0.10	0.36	−0.41	0.43	−0.07	−0.03	0.26	**0.25**
LG			0.09	0.36	−0.42	0.51	−0.07	0.06	0.27	**0.24**
P-ApG				0.59	0.00	0.29	0.14	−0.05	0.15	**0.11**
P-AvG					0.33	0.03	0.42	−0.02	−0.03	−**0.04**
WA						−0.41	0.22	0.08	-0.22	−**0.21**
HfS							−0.28	−0.03	0.33	**0.32**
FoF								−0.12	−0.27	−**0.28**
*g*									0.24	**0.28**
GPAt0										0.84
GPAt2										**—**

**N* = 345. ASC, ability self-concept; LG, learning goals; P-ApG, performance-approach goals; P-AvG, performance-avoidance goals; WA, work avoidance; HfS, hope for success; FoF, fear of failure; g, general intelligence; GPA, Grade Point Average; t0, before testing; t2, after testing. Correlations reflecting criterion-related validities are printed in bold. *r* ≥ ∣0.11∣, *p* < 0.05. *r* ≥ ∣0.14∣, *p* < 0.01. *r* ≥ ∣0.17∣, *p* < 0.001.*

**TABLE 3:** Intercorrelations between all variables in math.

**Motivation in math**	**Task values**	**LG**	**P-AgG**	**P-AvG**	**WA**	**HfS**	**FoF**	**Numerical**	**Math Gt0**	**Math Gt2**
ASC	0.76	0.57	0.54	0.21	−0.24	0.68	−0.42	0.36	0.68	**0.61**
Task values		0.70	0.60	0.25	−0.36	0.68	−0.32	0.21	0.54	**0.50**
LG			0.62	0.23	−0.45	0.64	−0.26	0.19	0.46	**0.42**
P-ApG				0.59	−0.14	0.52	−0.13	0.19	0.38	**0.28**
P-AvG					0.21	0.21	0.23	0.10	0.13	**0.07**
WA						−0.38	0.24	0.06	−0.29	−**0.34**
HfS							−0.35	0.28	0.51	**0.45**
FoF								−0.23	−0.30	−**0.30**
Numerical									−0.27	**0.22**
Math Gt0										**0.72**
Math Gt2										**—**

**N* = 345. ASC, ability self-concept; LG, learning goals; P-ApG, performance-approach goals; P-AvG, performance-avoidance goals; WA, work avoidance; HfS, hope for success; FoF, fear of failure; Numerical, numerical intelligence; Math G, math grade; t0, before testing; t2, after testing. Correlations reflecting criterion-related validities are printed in bold. *r* ≥ ∣0.11∣, *p* < 0.05. *r* ≥ ∣0.14∣, *p* < 0.01. *r* ≥ ∣0.17∣, *p* <0.001.*

**TABLE 4:** Intercorrelations between all variables in German.

**Motivation in German**	**Task values**	**LG**	**P-AgG**	**P-AvG**	**WA**	**HfS**	**FoF**	**Verbal**	**German G**	**German G**
ASC	0.68	0.58	−0.01	0.38	−0.36	0.55	−0.27	−0.17	0.41	**0.46**
Task Values		0.70	0.08	0.45	−0.37	0.58	−0.10	−0.21	0.30	**0.34**
LG			0.06	0.47	−0.47	0.65	−0.13	−0.12	0.34	**0.34**
P-ApG				0.55	−0.09	0.44	−0.01	−0.05	0.20	**0.23**
P-AvG					0.26	0.11	0.34	0.02	−0.01	−**0.03**
WA						−0.47	0.23	0.18	−0.20	−**0.21**
HfS							−0.30	−0.08	0.28	**0.33**
FoF								−0.16	−0.24	−**0.31**
Verbal									0.19	**0.10**
German Gt0										0.73
German Gt2										**—**

**N* = 345. ASC, ability self-concept; LG, learning goals; P-ApG, performance-approach goals; P-AvG, performance-avoidance goals; WA, work avoidance; HfS, hope for success; FoF, fear of failure; Verbal, verbal intelligence; German G, German grade; t0, before testing; t2, after testing. Correlations reflecting criterion-related validities are printed in bold. *r* ≥ ∣0.11∣, *p* < 0.05. *r* ≥ ∣0.14∣, *p* < 0.01. *r* ≥ ∣0.17∣, *p* < 0.001.*

### Relative Weight Analyses

Table 5 presents the results of the relative weight analyses. In Model 1 (only motivational variables) and Model 2 (motivation and intelligence), respectively, the overall explained variance was highest for math grades (*R*^2^ = 0.42 and *R*^2^ = 0.42, respectively) followed by GPA (*R*^2^ = 0.30 and *R*^2^ = 0.34, respectively) and grades in German (*R*^2^ = 0.26 and *R*^2^ = 0.28, respectively). When prior school grades were additionally considered (Model 3) the largest amount of variance was explained in students’ GPA (*R*^2^ = 0.73), followed by grades in German (*R*^2^ = 0.59) and math (*R*^2^ = 0.57). In the following, we will describe the results of Model 3 for each domain in more detail.

**TABLE 5:** Relative weights and percentages of explained criterion variance (%) for all motivational constructs (Model 1) plus intelligence (Model 2) plus prior school achievement (Model 3).

	**Model 1: Achievement motivation**						**Model 2: Achievement motivation + Intelligence**						**Model 3: Achievement motivation + Intelligence + Prior school achievement**					
		
**Predictor**	**RW**			**%**			**RW**			**%**			**RW**			**%**		
	**GPA**	**Math**	**German**	**GPA**	**Math**	**German**	**GPA**	**Math**	**German**	**GPA**	**Math**	**German**	**GPA**	**Math**	**German**	**GPA**	**Math**	**German**
Achievement t0													0.496^a^	0.259^a^	0.375^a^	68.3	45.3	64.1
Specific intelligence							0.059^b^	0.016^c^	0.035^b^	17.0	3.9	12.4	0.037^c^	0.012	0.022^c^	5.1	2.1	3.8
Ability self-concept	0.182^a^	0.172^a^	0.093^a^	60.0	41.1	35.9	0.170^a^	0.162^a^	0.088^a^	49.2	38.7	31.2	0.103^b^	0.106^b^	0.060^b^	14.2	18.5	10.3
Task Values	0.018^b^	0.067^b^	0.031^b^	5.9	16.1	11.9	0.021^c^	0.066^b^	0.031^b^	6.1	15.8	10.9	0.016^c^	0.053^c^	0.026^c^	2.2	9.3	4.4
Learning goals	0.014	0.038^b,c^	0.030^b^	4.7	9.1	11.7	0.013	0.037^b,c^	0.029^b^	3.7	8.9	10.3	0.011	0.031^c,d^	0.022^c^	1.5	5.4	3.8
P-ApG	0.005	0.016^d^	0.015	1.5	3.9	1.4	0.005	0.016^c^	0.015^c^	1.3	3.7	5.4	0.003	0.013	0.013	0.2	2.3	2.3
P-AvG	0.002	0.004	0.004	0.6	1.0	5.7	0.002	0.004	0.004	0.6	0.9	1.3	0.001	0.003	0.003	0.5	0.5	0.6
Work avoidance	0.011	0.047^b,c^	0.008	3.7	11.3	3.1	0.015	0.049^b^	0.009	4.3	11.7	3.2	0.011	0.038^c,d^	0.007	1.5	6.7	1.2
Hope for success	0.034^b^	0.047^b,c^	0.024^b^	11.4	11.2	9.2	0.031^b,c^	0.044^b^	0.025^b,c^	9.1	10.5	8.8	0.025^c^	0.036^c,d^	0.022^c^	3.5	6.2	3.8
Fear of failure	0.037^b^	0.027^c,d^	0.055^b^	12.3	6.4	21.2	0.030^b,c^	0.025^c^	0.047^b^	8.7	5.9	16.5	0.022^c^	0.020^d^	0.034^c^	3.1	3.6	5.7
Explained variance *R*^2^	0.303	0.418	0.259	100	100	100	0.344	0.419	0.284	100	100	100	0.726	0.572	0.585	100	100	100

*Criterion, students’ achievement after testing; GPA, Grade Point Average; P-ApG, performance-approach goals; P-AvG, performance-avoidance goals. Relative weights with a superscript are significantly different from zero at *p* < 0.05. Values with different superscripts are significantly different at *p* < 0.05.*

Beginning with the prediction of students’ GPA: In Model 3, students’ prior GPA explained more variance in subsequent GPA than all other predictor variables (68%). Students’ ability self-concept explained significantly less variance than prior GPA but still more than all other predictors that we considered (14%). The relative weights of students’ intelligence (5%), task values (2%), hope for success (4%), and fear of failure (3%) did not differ significantly from each other but were still significantly different from zero (*p* < 0.05). The relative weights of students’ goal orientations were not significant in Model 3.

Turning to math grades: The findings of the relative weight analyses for the prediction of math grades differed slightly from the prediction of GPA. In Model 3, the relative weights of numerical intelligence (2%) and performance-approach goals (2%) in math were no longer different from zero (*p* > 0.05); in Model 2 they were. Prior math grades explained the largest share of the unique variance in subsequent math grades (45%), followed by math self-concept (19%). The relative weights of students’ math task values (9%), learning goals (5%), work avoidance (7%), and hope for success (6%) did not differ significantly from each other. Students’ fear of failure in math explained the smallest amount of unique variance in their math grades (4%) but the relative weight of students’ fear of failure did not differ significantly from that of students’ hope for success, work avoidance, and learning goals. The relative weights of students’ performance-avoidance goals were not significant in Model 3.

Turning to German grades: In Model 3, students’ prior grade in German was the strongest predictor (64%), followed by German self-concept (10%). Students’ fear of failure in German (6%), their verbal intelligence (4%), task values (4%), learning goals (4%), and hope for success (4%) explained less variance in German grades and did not differ significantly from each other but were significantly different from zero (*p <* 0.05). The relative weights of students’ performance goals and work avoidance were not significant in Model 3.

## Discussion

In the present studies, we aimed to investigate the relative importance of several achievement motivation constructs in predicting students’ academic achievement. We sought to overcome the limitations of previous research in this field by (1) considering several theoretically and empirically distinct motivational constructs, (2) students’ intelligence, and their prior achievement, and (3) by assessing all predictors at the same level of specificity as the achievement criteria. We applied sophisticated statistical procedures to investigate the relations in three different domains, namely school in general, math, and German.

### Relative Importance of Achievement Motivation Constructs for Academic Achievement

Out of the motivational predictor variables, students’ ability self-concepts explained the largest amount of variance in their academic achievement across all sets of analyses and across all investigated domains. Even when intelligence and prior grades were controlled for, students’ ability self-concepts accounted for at least 10% of the variance in the criterion. The relative superiority of ability self-perceptions is in line with the available literature on this topic (e.g., Steinmayr and Spinath, 2009; Kriegbaum et al., 2015; Steinmayr et al., 2018) and with numerous studies that have investigated the relations between students’ self-concept and their achievement (e.g., Möller et al., 2009; Huang, 2011). Ability self-concepts showed even higher relative weights than the corresponding intelligence scores. Whereas some previous studies have suggested that self-concepts and intelligence are at least equally important when predicting students’ grades (e.g., Steinmayr and Spinath, 2009; Weber et al., 2013; Schneider et al., 2018), our findings indicate that it might be even more important to believe in own school-related abilities than to possess outstanding cognitive capacities to achieve good grades (see also Lotz et al., 2018). Such a conclusion was supported by the fact that we examined the relative importance of all predictor variables across three domains and at the same levels of specificity, thus maximizing criterion-related validity (see Baranik et al., 2010). This procedure represents a particular strength of our study and sets it apart from previous studies in the field (e.g., Steinmayr and Spinath, 2009). Alternatively, our findings could be attributed to the sample we investigated at least to some degree. The students examined in the present study were selected for the academic track in Germany, and this makes them rather homogeneous in their cognitive abilities. It is therefore plausible to assume that the restricted variance in intelligence scores decreased the respective criterion validities.

When all variables were assessed at the same level of specificity, the achievement motives hope for success and fear of failure were the second and third best motivational predictors of academic achievement and more important than in the study by Steinmayr and Spinath (2009). This result underlines the original conceptualization of achievement motives as broad personal tendencies that energize approach or avoidance behavior across different contexts and situations (Elliot, 2006). However, the explanatory power of achievement motives was higher in the more specific domains of math and German, thereby also supporting the suggestion made by Sparfeldt and Rost (2011) to conceptualize achievement motives more domain-specifically. Conceptually, achievement motives and ability self-concepts are closely related. Individuals who believe in their ability to succeed often show greater hope for success than fear of failure and vice versa (Brunstein and Heckhausen, 2008). It is thus not surprising that the two constructs showed similar stability in their relative effects on academic achievement across the three investigated domains. Concerning the specific mechanisms through which students’ achievement motives and ability self-concepts affect their achievement, it seems that they elicit positive or negative valences in students, and these valences in turn serve as simple but meaningful triggers of (un)successful school-related behavior. The large and consistent effects for students’ ability self-concept and their hope for success in our study support recommendations from positive psychology that individuals think positively about the future and regularly provide affirmation to themselves by reminding themselves of their positive attributes (Seligman and Csikszentmihalyi, 2000). Future studies could investigate mediation processes. Theoretically, it would make sense that achievement motives defined as broad personal tendencies affect academic achievement via expectancy beliefs like ability self-concepts (e.g., expectancy-value theory by Eccles and Wigfield, 2002; see also, Atkinson, 1957).

Although task values and learning goals did not contribute much toward explaining the variance in GPA, these two constructs became even more important for explaining variance in math and German grades. As Elliot (2006) pointed out in his hierarchical model of approach-avoidance motivation, achievement motives serve as basic motivational principles that energize behavior. However, they do not guide the precise direction of the energized behavior. Instead, goals and task values are commonly recruited to strategically guide this basic motivation toward concrete aims that address the underlying desire or concern. Our results are consistent with Elliot’s (2006) suggestions. Whereas basic achievement motives are equally important at abstract and specific achievement levels, task values and learning goals release their full explanatory power with increasing context-specificity as they affect students’ concrete actions in a given school subject. At this level of abstraction, task values and learning goals compete with more extrinsic forms of motivation, such as performance goals. Contrary to several studies in achievement-goal research, we did not demonstrate the importance of either performance-approach or performance-avoidance goals for academic achievement.

Whereas students’ ability self-concept showed a high relative importance above and beyond intelligence, with few exceptions, each of the remaining motivation constructs explained less than 5% of the variance in students’ academic achievement in the full model including intelligence measures. One might argue that the high relative importance of students’ ability self-concept is not surprising because students’ ability self-concepts more strongly depend on prior grades than the other motivation constructs. Prior grades represent performance feedback and enable achievement comparisons that are seen as the main determinants of students’ ability self-concepts (see Skaalvik and Skaalvik, 2002). However, we included students’ prior grades in the analyses and students’ ability self-concepts still were the most powerful predictors of academic achievement out of the achievement motivation constructs that were considered. It is thus reasonable to conclude that the high relative importance of students’ subjective beliefs about their abilities is not only due to the overlap of this believes with prior achievement.

### Limitations and Suggestions for Further Research

Our study confirms and extends the extant work on the power of students’ ability self-concept net of other important motivation variables even when important methodological aspects are considered. Strength of the study is the simultaneous investigation of different achievement motivation constructs in different academic domains. Nevertheless, we restricted the range of motivation constructs to ability self-concepts, task values, goal orientations, and achievement motives. It might be interesting to replicate the findings with other motivation constructs such as academic self-efficacy (Pajares, 2003), individual interest (Renninger and Hidi, 2011), or autonomous versus controlled forms of motivation (Ryan and Deci, 2000). However, these constructs are conceptually and/or empirically very closely related to the motivation constructs we considered (e.g., Eccles and Wigfield, 1995; Marsh et al., 2018). Thus, it might well be the case that we would find very similar results for self-efficacy instead of ability self-concept as one example.

A second limitation is that we only focused on linear relations between motivation and achievement using a variable-centered approach. Studies that considered different motivation constructs and used person-centered approaches revealed that motivation factors interact with each other and that there are different profiles of motivation that are differently related to students’ achievement (e.g., Conley, 2012; Schwinger et al., 2016). An important avenue for future studies on students’ motivation is to further investigate these interactions in different academic domains.

Another limitation that might suggest a potential avenue for future research is the fact that we used only grades as an indicator of academic achievement. Although, grades are of high practical relevance for the students, they do not necessarily indicate how much students have learned, how much they know and how creative they are in the respective domain (e.g., Walton and Spencer, 2009). Moreover, there is empirical evidence that the prediction of academic achievement differs according to the particular criterion that is chosen (e.g., Lotz et al., 2018). Using standardized test performance instead of grades might lead to different results.

Our study is also limited to 11th and 12th graders attending the highest academic track in Germany. More balanced samples are needed to generalize the findings. A recent study (Ben-Eliyahu, 2019) that investigated the relations between different motivational constructs (i.e., goal orientations, expectancies, and task values) and self-regulated learning in university students revealed higher relations for gifted students than for typical students. This finding indicates that relations between different aspects of motivation might differ between academically selected samples and unselected samples.

Finally, despite the advantages of relative weight analyses, this procedure also has some shortcomings. Most important, it is based on manifest variables. Thus, differences in criterion validity might be due in part to differences in measurement error. However, we are not aware of a latent procedure that is comparable to relative weight analyses. It might be one goal for methodological research to overcome this shortcoming.

## Conclusion

We conducted the present research to identify how different aspects of students’ motivation uniquely contribute to differences in students’ achievement. Our study demonstrated the relative importance of students’ ability self-concepts, their task values, learning goals, and achievement motives for students’ grades in different academic subjects above and beyond intelligence and prior achievement. Findings thus broaden our knowledge on the role of students’ motivation for academic achievement. Students’ ability self-concept turned out to be the most important motivational predictor of students’ grades above and beyond differences in their intelligence and prior grades, even when all predictors were assessed domain-specifically. Out of two students with similar intelligence scores, same prior achievement, and similar task values, goals and achievement motives in a domain, the student with a higher domain-specific ability self-concept will receive better school grades in the respective domain. Therefore, there is strong evidence that believing in own competencies is advantageous with respect to academic achievement. This finding shows once again that it is a promising approach to implement validated interventions aiming at enhancing students’ domain-specific ability-beliefs in school (see also Muenks et al., 2017; Steinmayr et al., 2018).

## Data Availability

The datasets generated for this study are available on request to the corresponding author.

## Ethics Statement

In Germany, institutional approval was not required by default at the time the study was conducted. That is, why we cannot provide a formal approval by the institutional ethics committee. We verify that the study is in accordance with established ethical guidelines. Participation was voluntarily and no deception took place. Before testing, we received informed consent forms from the parents of the students who were under the age of 18 on the day of the testing. If students did not want to participate, they could spend the testing time in their teacher’s room with an extra assignment. All students agreed to participate. We included this information also in the manuscript.

## Author Contributions

RS conceived and supervised the study, curated the data, performed the formal analysis, investigated the results, developed the methodology, administered the project, and wrote, reviewed, and edited the manuscript. AW wrote, reviewed, and edited the manuscript. MS performed the formal analysis, and wrote, reviewed, and edited the manuscript. BS conceived the study, and wrote, reviewed, and edited the manuscript.

## Conflict of Interest Statement

The authors declare that the research was conducted in the absence of any commercial or financial relationships that could be construed as a potential conflict of interest.
